# MDSVDNV: predicting microbe–drug associations by singular value decomposition and Node2vec

**DOI:** 10.3389/fmicb.2023.1303585

**Published:** 2024-01-08

**Authors:** Huilin Tan, Zhen Zhang, Xin Liu, Yiming Chen, Zinuo Yang, Lei Wang

**Affiliations:** Big Data Innovation and Entrepreneurship Education Center of Hunan Province, Changsha University, Changsha, China

**Keywords:** microbe–drug association prediction, computational model, singular value decomposition, Node2vec, XGBoost classifier

## Abstract

**Introduction:**

Recent researches have demonstrated that microbes are crucial for the growth and development of the human body, the movement of nutrients, and human health. Diseases may arise as a result of disruptions and imbalances in the microbiome. The pathological investigation of associated diseases and the advancement of clinical medicine can both benefit from the identification of drug-associated microbes.

**Methods:**

In this article, we proposed a new prediction model called MDSVDNV to infer potential microbe-drug associations, in which the Node2vec network embedding approach and the singular value decomposition (SVD) matrix decomposition method were first adopted to produce linear and non-linear representations of microbe interactions.

**Results and discussion:**

Compared with state-of-the-art competitive methods, intensive experimental results demonstrated that MDSVDNV could achieve the best AUC value of 98.51% under a 5-fold CV, which indicated that MDSVDNV outperformed existing competing models and may be an effective method for discovering latent microbe–drug associations in the future.

## Introduction

The microbial community is crucial for both health and disease. It contains bacteria, archaea, viruses, protozoa, and fungi that are present in various organs of the human body and may be deficient in beneficial functions as well as harmful functions (Ventura et al., 2009). Therefore, the imbalances in the composition of the microbial community may lead to several diseases (Kashyap et al., 2017). For instance, obesity and inflammatory diseases might result from a lack of microbial diversity (Huttenhower et al., 2012), and the higher microbial diversity in the vagina is associated with bacterial vaginal diseases (Huang et al., 2017). Thus, repairing missing beneficial functions and eliminating harmful microbial activity functions could help in treating certain diseases. Many possible human microbe–drug connections are yet to be uncovered, and the mechanism of association between bacteria and medication has only received a limited amount of research; however, in practice, antibiotic treatment of microbial communities produces some collateral damage, traditional clinical trials are tedious and costly, and it can sometimes take at least 10 years for a novel therapy to reach the market. Moreover, from lab research to the market, a new drug might cost up to a billion dollars (Adams and Brantner, 2006; Cummings et al., 2018). Currently, since the known associations between microbes and drugs are very limited, and it is quite expensive and time-consuming to discover them through a large number of experiments conducted by medical means, it will save plenty of time and money to predict the potential associations between them based on computational models and then verify them through medical experiments. In addition, the discovery of latent associations between human microorganisms, drugs, and diseases can provide further understanding of the potential mechanism of disease occurrence from the perspective of human microorganisms and drugs, so as to provide great help for the study of pathogenesis, facilitate early diagnosis, and improve the precision of medication.

Due to the rapid expansion of computer storage and processing capacity in recent years, a large collection of biological databases of related microbes and drugs, such as MDAD, aBiofilm, and DrugVirus, as well as HDVD and the COVID-19 database, have been established successfully, based on which it becomes possible to adopt machine learning techniques to infer new microbe–drug interactions. For instance, Zhu et al. proposed a computational model HMDAKATZ based on the KATZ measure by fusing the chemical structure-similarity of pharmaceuticals with the GIP nuclear similarity of microbes to infer potential microbe–drug associations (Zhu et al., 2019). Dong et al. proposed a method called HNERMDA by incorporating a network embedding technique called metapath2ve with a two-part network recommendation algorithm to detect latent associations between microbes and drugs (Dong et al., 2017). Although the KATZ measurement can simultaneously reconstruct potential associations in large-scale networks, the similarity will inevitably be biased toward those known associations when calculating the GIP kernel similarity. Different from KATZ, HeteSim is a general framework for correlation metrics in heterogeneous networks that can efficiently capture the subtle semantics of search paths. Shi et al. proposed a HeteSim-based method for relevance measure in heterogeneous networks, which can effectively capture potential subtle semantic associations but cannot accomplish the prediction of microorganisms (drugs and diseases) without any known association (Shi et al., 2014). Therefore, for the past few years, scholars have introduced matrix completion and matrix decomposition to break down missing value matrices into two or more separate matrices first, and then these matrices will be multiplied to provide an approximation of the original matrix. As a result, in 2018, Shi et al. introduced a prediction model called BMCMDA based on the completion of binary matrices (Shi et al., 2018), which involves complex singular value decomposition. Zhu et al. suggested a fresh computational technique named LRLSMDA based on the Laplacian regularized least square algorithm by using the minimization of the cost function to compute the two objective functions and transforming them into the prediction matrices using the linear averaging method (Zhu et al., 2021). In 2022, Cheng et al. proposed a computational model NIRBMMDA based on neighborhood reasoning and restricted Boltzmann machines, which searches for similar neighbors of drugs or microbes through different thresholds to obtain a scoring matrix of potential microbe–drug associations (Cheng et al., 2022). In comparison to existing methods, this sort of regularization method generates fewer model parameters, which saves time and improves robust performance. It also aims to build different regularized least squares classifications (a squared loss regularization network with a kernel) to resolve various prediction problems. Whereas, the later emergence of neural networks is considered a revolutionary change and performs well in the direction of biological prediction. For example, Huang et al. presented a prediction model GNAEMDA based on graph normalized convolutional networks, which constructs a multimodal attribute map by collecting features, then inputs them into a graph normalized convolutional network, and finally uses the reconstructed map output from the network to make unknown association predictions (Huang et al., 2023). Additionally, Huang et al. designed a prediction model called Graph2MDA based on the variational graph autoencoder (VGAE) (Deng et al., 2021), which develops a deep neural network-based classifier to infer potential microbe–drug associations by using a two-layer graph convolutional network (GCN)-based encoder to train low-dimensional representations. In 2020, Long et al. proposed a calculation model named GCNMDA by combining a GCN-based encoder with a CRF layer and a decoder to forecast potential microbe–disease associations (Long et al., 2020). At present, attention mechanisms have been widely used to increase the effect of important data points, based on which graph attention network (GAT)-based encoders have been popular in recent years for biological prediction. For instance, Long et al. introduced an integrated GAT framework named EGATMDA (Long et al., 2020), which includes two attention mechanisms and three kinds of networks such as the microbe–drug two-part network, the microbe–drug heterogeneous network, and the microbe–disease–drug heterogeneous network. Later, in order to ensure the sparsity of the hidden layer, the sparse autoencoder (SAE) added a penalty clause to the autoencoder. For example, Jiang et al. designed a novel approach called SAEROF to predict potential disease–drug associations by utilizing the SAE and the principal component analysis (PCA) for feature extraction and a rotating forest classifier for the final prediction (Jiang et al., 2020). Since one of the fundamental tasks in the field of bioinformatics is to forecast possible associations between biological entities, researchers not only have produced excellent results in the field of microbe–drug association prediction but also have developed a wealth of wonderful techniques for the microbe–disease association prediction, the virus–drug association prediction, the circRNAs–disease association forecasting, and the interactions forecasting between molecules and miRNAs. For instance, Qu et al. introduced a calculation model MHBVDA to predict antiviral drugs based on both heterogeneous graphical inference matrix decomposition and bounded kernel paradigm regularization (Qu et al., 2023). In 2023, Wang et al. designed a prediction method TNRGCN for microbe–disease association prediction based on a tripartite network of microbes–drugs–diseases and a relational graph convolutional network (RGCN) (Wang et al., 2023). Chen presented a detection model MATHMDA by integrating meta-path aggregate graph neural networks and heterogeneous networks to infer latent relationships between microbes and diseases (Chen and Lei, 2022). In addition, Peng et al. created a prediction model named GATCL2CD by assessing similarities between circRNAs and diseases, in which a heterogeneous network was built first, and then, based on the heterogeneous network, a graph attention network for feature convolution learning was proposed to predict circRNA disease connections (Peng et al., 2023). Additionally, Peng et al. employed a scalable tree-enhanced model to predict potential correlations between each pair of small-molecule miRNAs, in which a deep autoencoder was adopted to produce probable feature representations of each pair (Peng et al., 2022).

Although the above models performed reliably in some aspects, there are still some limitations. Taking neural networks widely used in the field of prediction as an example, the pooling layer of a convolutional neural network (CNN) will lose a lot of valuable information, which would result in a decline in the resolution of the output features and a decrease in the predictive ability of the model (Min et al., 2021). In addition, although GCN can improve the inapplicability of translation invariance to non-matrix structures it cannot learn better representative sample features through the convolution operation of the graph Laplacian-based structure information and input sample information (Sichao et al., 2021). As for GAT and SAE, GAT can effectively enhance the aggregation of graph neural networks, but it is difficult to aggregate higher-order objects and is sensitive to parameter initialization. SAE can extract abstract features of lower dimensionality and sparsity, but it cannot specify whether a node is active or hidden, and in addition, the sparsity parameter setting is poor (Wang et al., 2022). Inspired by the successful application of network embedding and matrix decomposition methods in the field of bioinformatics, in this article, singular value decomposition and Node2Vec are integrated into the prediction model MDSVDNV to infer potential microbe–drug correlations. The prediction performance on datasets of different scales shows that MDSVDNV can adapt to a large range of datasets with strong robustness. In MDSVDNV, we first extracted the linear feature representations of the interactions between microbes and drugs based on the matrix decomposition approach of singular value decomposition. Then, we acquired the network topological information-containing non-linear features between microbes and drugs via the node2vec algorithm. Finally, we fused its linear and non-linear features to form an integrated feature vector for each microbe and drug and inputted these integrated feature vectors into XGBoost, a machine learning classifier, to gain the anticipated scores of potential microbe–drug associations and convert the microbe–drug association prediction issue to a binary classification problem while predicting potential correlations between microbes and drugs.

Compared with state-of-the-art competitive methods, intensive experimental results demonstrated that MDSVDNV could achieve the best AUC value of 98.51% under the 5-fold CV, which indicated that MDSVDNV is superior to existing competing models and may play an important role in predicting potential microbe–drug associations in the future. The main contributions to this article are as follows:

∙MDSVDNV can be regarded as an open framework in which more feature extraction methods can be applied flexibly for the fusion of linear and non-linear features.

∙MDSVDNV is able to adapt to a large range of datasets, since it is robust and less time-consuming.

## Materials and methods

### Datasets

In experiments, we first downloaded known microbe–drug associations from the MDAD database (https://figshare.com/search?q=10.6084%2Fm9.figshare.24798456) and the aBiofilm database (https://bioinfo.imtech.res.in/manojk/abiofilm/) separately. As a result, we downloaded obtained 5,505 clinically reported or experimentally validated microbial–drug correlations between 1,388 drugs and 174 microorganisms collected in 993 articles from the MDAD database, and after excluding duplicates, we obtained a microbial–drug dichotomous network containing 1,373 drugs and 173 microorganisms, and 2,470 relationships between 1,373 drugs and 140 microorganisms, while 2,884 known microbe–drug associations between 1720 drugs and 140 microorganisms were obtained from the aBiofilm database. Based on these newly downloaded known microbe–drug associations, we created an adjacency matrix *A*∈ $R^{n_{r}*n_{m}}$ as follows: If there is a known link between the drug $r_{i}$ and the microbe $m_{j}$ , then there is $A_{ij}$ =1; otherwise, there is $A_{ij}$ =0. Here, $n_{r}$ and $n_{m}$ denote the number of medicines and microorganisms, respectively.

### Our MDSVDNV model

**Figure 1:**
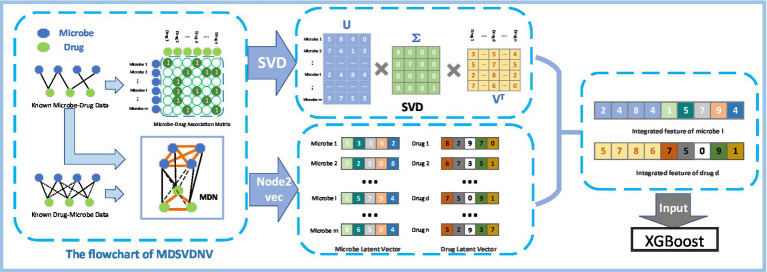
Flowchart of MDSVDNV.

Figure 1 illustrates the flowchart of the MDSVDNV, which consists of the following five major steps :

- **Step1**: Constructing the microbe–drug association matrix and corresponding microbe–drug association network (MDN).
- **Step2**: Applying the singular value decomposition, a matrix decomposition method, on the microbe–drug association matrix to extract the linear features of microbes and drugs.
- **Step3**: Applying Node2vec, a network embedding method, to the microbe–drug–disease association network to obtain the non-linear features of microbes and drugs.
- **Step4**: Fusing the linear and non-linear features of each microbe and drug to construct an integrated feature vector.
- **Step5**: The predicted scores of probable connections between microbes and drugs are obtained by feeding all these integrated feature vectors into the XGBoost machine learning classifier.

### Linear feature extraction based on the singular value decomposition (SVD)

One of the most prevalent algorithms in recommendation systems is matrix decomposition (Ma and Liu, 2022). As a standard recommendation system for collaborative filtering based on SVD (Vozalis and Margaritis, 2007), the idea of SVD is to transform an arbitrary matrix $A_{M\times N}$ into *A*= $U\Sigma V^{T}$ by a set of orthogonal basis transformations. Typically, as illustrated in the following Equation (1), three matrices are combined to decompose a matrix in SVD:


(1) $$A_{M\times N}=U_{M\times C}\cdot \Sigma_{C\times C}\cdot V_{C\times N}^{T}$$

Based on the Equation (2), the singular values in the matrix Σ are arranged as follows:


(2) $$\Sigma =\left[\begin{array}{c} \begin{array}{cc} \lambda_{1} & 0\\ 0 & \ddots \end{array}\;\begin{array}{cc} \ldots & 0\\ 0 & 0 \end{array}\\ \begin{array}{cc} \vdots & 0\\ 0 & \ldots \end{array}\;\begin{array}{cc} \lambda_{r} & \vdots \\ \ldots & 0 \end{array} \end{array}\right]$$

In Equations (2), supposing that there is $\lambda_{1}$ ≥ $\lambda_{2}$ ≥ … ≥ $\lambda_{r}$ , and $\lambda_{r}$ ≥0 (*i* = 1, 2, …, *r*) is the singular value of the matrix Σ . It is well known that the magnitude of the singular values indicates the importance of the corresponding vector; moreover, the singular values are arranged in descending order; the singular values at the top must reflect the original data better than the singular values at the bottom; especially, the singular values decay exceptionally fast from the largest to the smallest; and in most cases, the sum of the top 10% or even 1% of the singular values exceeds 99% of the sum of all the singular values (Wu et al., 2019). This is one of the principles of SVD data compression, which can handle large-scale data very well. Similarly, applying the singular value decomposition to the microbe–drug association matrix $A_{M\times N}$ yields the matrices $U,\;\Sigma ,\;V^{T}$ representing the microbe feature matrix, the matrix of feature weights, and the drug feature matrix, respectively. Especially in the microbe–drug association prediction problem, the most useful information about the microbe and drug features in a biomedical sense will be contained in the first 10% or even less of the singular values. During the dimensional reduction process, the useful data will not be lost, but the redundant information will be discarded. That is, we can obtain an approximate representation of the matrix *A* by keeping the *k* largest singular values based on the Equation (3):


(3) $$A_{M\times N}\approx U_{M\times K}\cdot \Sigma_{K\times K}\cdot V_{K\times N}^{T}$$

**Figure 2:**
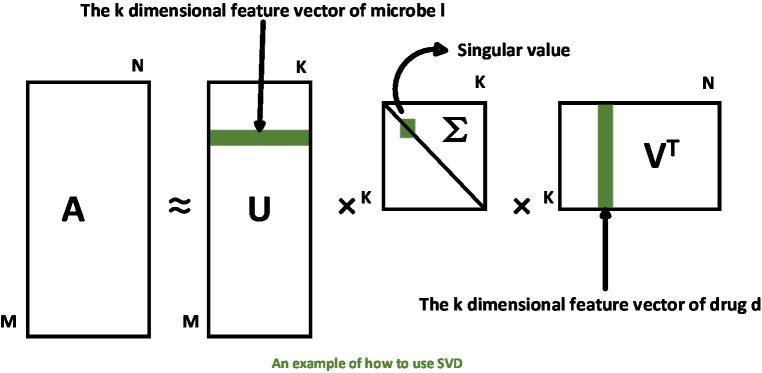
Example of how to use SVD on the microbe–drug relationship matrix.

We draw an example of SVD as in Figure 2. It is obvious that, based on the singular value decomposition method, each row in $U_{M\times K}$ represents a microbe’s *k*-dimensional linear feature vector. Similarly, each column in $V_{K\times N}^{T}$ represents a drug’s k-dimensional linear feature vector.

### Node2vec-based non-linear feature extraction

In order to train our model more accurately and realistically, we employed an accurate and sophisticated network embedding method called Node2vec to capture the mapping of microbe and drug nodes in low-dimensional space to features in low-dimensional space while maximizing the possibility of preserving network properties. Node2vec is a semi-supervised method for representing feature embeddings of nodes in a network (Grover and Leskovec, 2016). The algorithm is an innovative stochastic wandering by adjusting two parameters, p and q, so that the randomly sampled node moves to the next node with bias, unlike the traditional unbiased stochastic wandering in the past, which explored the neighborhoods of both breadth-first sampling and depth-first sampling. Node2vec generates the feature vectors of the nodes by using the Skip-gram model (Mikolov et al., 2013a), a word embedding approach that seeks to classify a word as accurately as possible based on other words in the same phrase and learns distributed vector representations from a huge text corpus. In reality, each node in the sequence of nodes produced by a biased random walk algorithm represents a word. The sequence encoding of nodes serves as the input of the model, and the nodes before and after the sequence serve as its output. We kept all the original parameter settings and extracted 16, 32, 64, 128, and 256 dimensions in our experiments. The experimental results showed that 256 dimensions would make the whole evaluation index higher, so we set the dimensions to 256 dimensions.

### Biased random wandering sampling strategy

**Figure 3:**
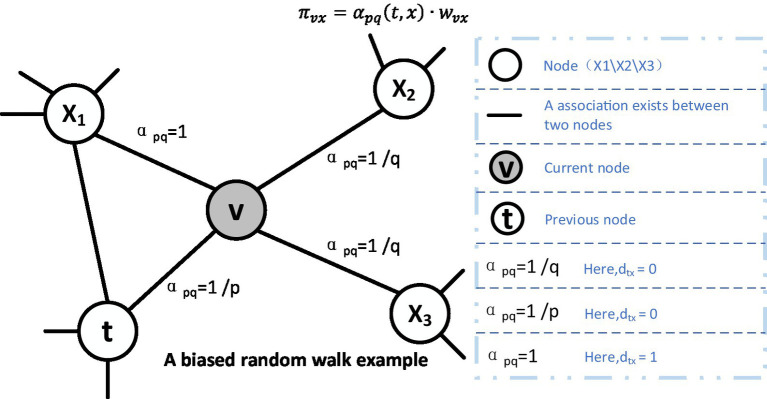
Illustration of the random walk procedure in node2vec.The walk just transitioned from *t* to *v* and is now evaluating its next step out of node *v*. Edge labels indicate search biases α.

For random wandering, we illustrated a schematic diagram in Figure 3, in which, supposing that the node *v* is the current node and its previous node is the node *t*. Then, as illustratred in the Equation (4), the next node will be selected based on the following static edge weights:


(4) $$\pi_{vx}=\alpha_{pq}\left(t,x\right)\cdot w_{vx}$$

where $w_{vx}$ is the static edge weights and $\pi_{vx}$ is the non-normalized transition deflection degree on the edge (*v*, *x*) induced by *v*, which will be affected by the weight tuning parameter *α*. When the number of nodes in the sequence reaches the predetermined walk length *l*, the algorithm will be ended. In addition, the parameter α is defined in the following Equation (5):


(5) $$\alpha_{pq}\left(t,\;x\right)=\{\begin{array}{c} \frac{1}{p}\;\mathrm{if}\;d_{tx}=0\\ 1\;\mathrm{if}\;d_{tx}=1\\ \frac{1}{q}\;\mathrm{if}\;d_{tx}=2 \end{array}$$

where the value of $d_{tx}$ might be either 0 or the shortest distance between *t* and *v*, and the shortest distance between t and *v* might be 1 or 2. The value of q determines whether to favor the breadth-first sampling or the depth-first sampling, and the value of *p* governs the deflection degree that the next walk will return to the previous node; if *p* is greater than 1, the random walk will have a reduced inclination to return, which guides the deviation of the random walk; if *q* is higher, the random walk will deviate less frequently. *p* is also the return parameter, which controls the deflection degree of returning to the original node. In summary, the settings of *p* and *q* can be summarized in the following way:

1. When $d_{tx}$ = 0, then the random walk will return from *x* to *t*. Since the search bias at this point is 1/*p*, going back to the previous step has a chance of 1/*p*.
2. When $d_{tx}$ = 1, then *x* is a direct neighbor of *t*, and the deviation is 1.
3. When $d_{tx}$ = 2, then *x* is a neighbor of *t*, and the deviation is 1/*q*.

The Node2vec stochastic process refers to the biased random wandering mentioned above, where the machine randomly selects sequences and learns their vectorial features in a fixed step size set in advance. In experiments, we will adopt the original parameter choices by setting *p* to 0.25 and *q* to 4.

### Node2vec feature learning implementation

In our method, we will transform the problem of extracting the characteristics of any given node in a network into a problem of optimizing an objective function of “likelihood” so that the node can save information about its neighbors. Hence, in the graph *G* = (*V*, *E*), where *V* is the set of nodes and *E* is the set of edges, as illustrated in the Equation (6), the objective function for maximizing the logarithmic property can be described as follows:


(6) $$\max_{f}\underset{v\in V}{\sum }\log\mathrm{Pr}\left(N_{s}\left(v\right)|f\left(v\right)\right)$$

where the function f : v → $R^{d}$ represents the mapping of vertices to feature representations, and *d* is a preset hyper-parameter indicating the dimension of the feature representation of each vertex. Thus, *f* is a function of size |*V*| × *d*, v∈V , and $N_{s}\left(v\right)\subset V$ denotes the neighboring vertices of vertex *v* under the sampling strategy *s.*

During implementation, we will apply the following two common conditional independence assumptions to make the optimization issue tractable:

·The assumption of conditional independence: In order to decompose the conditional probability, as shown in the Equation (7), we assume that, given the feature representation of the source node, the probability of the occurrence of its nearest-neighbor vertices is independent of the remaining vertices in the nearest-neighbor set, which can be represented as:


(7) $$\mathrm{Pr}\left(N_{s}\left(v\right)|f\left(v\right)\right)=\underset{n_{i}\in N_{s}\left(v\right)}{\prod }\mathrm{Pr}\left(n_{i}|f\left(v\right)\right)$$

·Symmetry in feature space: Considering that the source node and its neighbor nodes have a symmetry effect on each other in the feature space, which means that a vertex shares the same set of embedding vectors as a source vertex and as a near-neighbor vertex; therefore, by parameterizing each source-neighborhood node pair as a softmax unit, as illustrated in the Equation (8), we can model the conditional probability as follows:


(8) $$\mathrm{Pr}\left(n_{i}|f\left(v\right)\right)=\frac{\exp\left(f\left(n_{i}\right)\cdot f\left(v\right)\right)}{\sum_{x\in V}\exp\left(f\left(x\right)\cdot f\left(v\right)\right)}$$

The above two assumptions aim to help with the optimization challenge and allow for the objective function to be simplified in the manner shown in the following Equation (9):


(9) $$\max_{f}\underset{v\in V}{\sum }\left[-\log Z_{v}+\underset{u_{i}\in N_{s}\left(v\right)}{\sum }f\left(v_{i}\right)\cdot f\left(v\right)\right]$$

For each node, as illustrated in the Equation (10), there are:


(10) $$Z_{v}=\underset{x\in V}{\sum }\exp\left(f\left(v\right)\cdot f\left(x\right)\right)$$

For large networks, the matching function $Z_{v}=\underset{x\in V}{\sum }\exp\left(f\left(v\right)\cdot f\left(x\right)\right)$ at each node is computationally expensive, and we use negative sampling (Mikolov et al., 2013b) to approximate it. It is a method used to increase the training speed and improve the quality of the resulting feature vectors. A small, randomly selected negative sample is used to update the corresponding weights. Unlike the original method, where all the weights are updated for each training sample, negative sampling allows a training sample to be updated with only a small portion of the weights at a time, which reduces the amount of computation in the gradient descent process.

### Feature fusion

In the microbe–drug association network, we get the $U,V^{T}$ , and non-linear feature representations of the disease and microbe nodes based on the decomposition of $A_{M\times N}$ and the semi-supervised algorithm Node2vec. The following is the feature fusion rule for each microbe *i* and drug *j*: The *i* th row of *U*, which is transformed into a column vector and given the symbol $LM_{i}$ , is the linear feature corresponding to the microbe *i*. Similar to this, the *j*th column of $V^{T}$ , designated as $LD_{i}$ , is the linear features related to the drug *j*. In addition, after designating the non-linear feature corresponding to *i* as $NM_{j}$ and the non-linear feature relating to j as $ND_{j}$ , as shown in the Equations (11) and (12), the combined final features of the nodes *i* and *j* can be featured as follows:


(11) $$FM_{i}=\left[\begin{array}{c} LM_{i}\\ NM_{i} \end{array}\right]$$

(12) $$FD_{j}=\left[\begin{array}{c} LD_{j}\\ ND_{j} \end{array}\right]$$

where [] denotes a vector connection operation.

Ultimately, we use the final combined features of drugs and microbes as the input to the XGBoot classifier, which converts the prediction task to a binary classification task, and the output of the XGBoot classifier yields a linear relationship (i.e., association probability) between each pair of microbes and drug, thus we can determine the hidden associations between microbes and drugs. In experiments, we finally set max depth = 2, min child weight = 50, subsample = 0.3, and the remaining parameters to their default values.

### Evaluation metrics

As in most other works, we performed 5-fold cross-validation to evaluate the performance of MDSVDNV, and in experiments, we adopted five kinds of evaluation metrics such as the true positive rate (TPR), false positive rate (FPR), accuracy, and recall associated with the ROC and PR curves, which were defined in the following Equations (13), (14), (15), (16) and (17):


(13) $$TPR=\frac{TP}{TP+FN}$$

(14) $$FPR=\frac{FP}{TN+FP}$$

(15) $$\mathrm{Precision}=\frac{TP}{TN+FP}$$

(16) $$\mathrm{Recall}=\frac{TP}{TP+FN}$$

(17) $$\mathrm{Accuracy}=\frac{TP+TN}{TP+TN+FP+FN}$$

where TP and TN denote the number of correctly predicted positive and negative samples, respectively, and FN and FP represent the number of incorrectly identified positive and negative samples, respectively.

## Results

In order to validate the capability of MDSVDNV, we conducted intensive experiments under the framework of *k*-fold cross-validation to compare the performance of MDSVDNV with existing state-of-the-art prediction models. Experimental results show that MDSVDNV outperformed all these competing methods. Additionally, we further performed ablation experiments under quintuple cross-validation to verify whether the combination of linear and non-linear features would favor the predictive ability of MDSVDNV. Finally, case studies of two commonly used antimicrobial drugs and a microorganism have demonstrated the effectiveness of MDSVDNV in real-world applications as well, which means that MDSVDNV can achieve acceptable predictive performance and may be a useful method for revealing potential microbe–drug interactions in the future.

### Performance comparison with other algorithms

In this section, we will compare MDSVDNV with a few representative methods for the association prediction problem. Since microbial–drug association prediction is a novel problem, there are currently few available computational methods and codes. In addition, we will also compare MDSVDNV with four state-of-the-art microbe–drug association prediction models, including HMDAKATZ (Zhu et al., 2021), LAGCN (Yu et al., 2020), NTSHMDA (Luo and Long, 2020), and BPNNHMDA (Li et al., 2021), utilizing the 5-fold CV. There will be no overlap between the training set and the test set, and each sample can be examined by our model through the 5-fold CV, in which the average training loss and the average validation loss of the five models are taken to measure the advantages and disadvantages of hyperparameters. In the 5-fold CV, we will randomly divide all microbe–drug susceptibility correlations into five equal parts, each as a test set, and the remaining four as the training set. After eventually finding a suitable hyper-parameter, we will train one model using the entire set of data as the hyper-parameter. The performance of the approach is then measured by plotting the receiver operating characteristic (ROC) curve and determining the area under the ROC curve (AUC). Overall, the higher the AUC value, the better the prediction performance, and an AUC value less than 0.5 indicates a strong random classification ability. Among them, HMDAKATZ is a KATZ-based microbial-drug association prediction method; NTSHMDA is a random walk and restart-based model designed to detect potential microbial–disease associations; and BPNNHMDA is a neural network-based model designed to infer potential microbe–disease associations. LAGCN is a graph convolutional network and attentional mechanism-based method. The aforementioned models will go through a 5-fold CV test based on MDAD and aBiofilm for a fair comparison. Additionally, although the aforementioned models employ several assessment metrics, we only use AUC, AUPRC, and accuracy values to assess how well these models predict outcomes in this section. Therefore, Table 1 lists the AUC and AUPRC values as well as the accuracy values for MDSVDNV, HMDAKATZ, LAGCN, NTSHMDA, and BPNNHMDA. From Table 1, it can be seen that MDSVDNV can obtain the best AUC and AUPRC values at the same time.

**Table 1:** AUCs, AUPRCs, and accuracy of compared methods based on datasets MDAD and aBiofilm under a 5-fold CV.

Methods	AUC(%)		AUPRC(%)		Accuracy(%)	
	MDAD	aBiofilm	MDAD	aBiofilm	MDAD	aBiofilm
LAGCN	0.8533 ± 0.0070	0.8641 ± 0.0109	0.3571 ± 0.0051	0.3671 ± 0.0055	0.9413	0.9373
NTSHMDA	0.8483 ± 0.0020	0.8610 ± 0.0022	0.1892 ± 0.0056	0.1962 ± 0.0078	**0.9896**	**0.9882**
HMDAKATZ	0.8712 ± 0.0010	0.8993 ± 0.0021	0.2327 ± 0.0068	0.3066 ± 0.0077	0.9774	0.9796
BPNNHMDA	0.8410 ± 0.0320	0.8438 ± 0.0186	0.0391 ± 0.0105	0.0476 ± 0.0067	0.9894	0.9869
MDSVDNV	**0.9851 ± 0.0034**	0.9875 ± 0.0045	**0.9731 ± 0.0070**	0.9763 ± 0.0053	0.9434	0.9401

**Figure 4:**
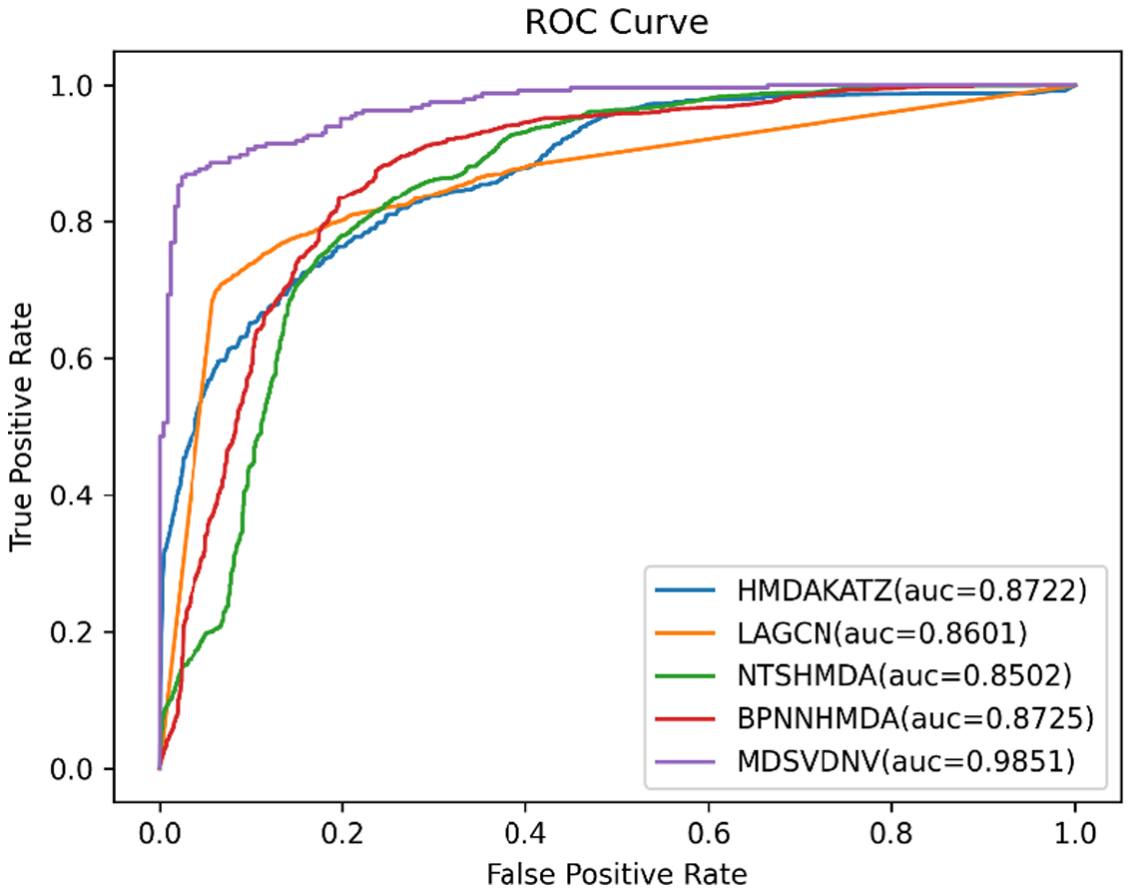
ROC curves of five competitive methods on MDAD.

**Figure 5:**
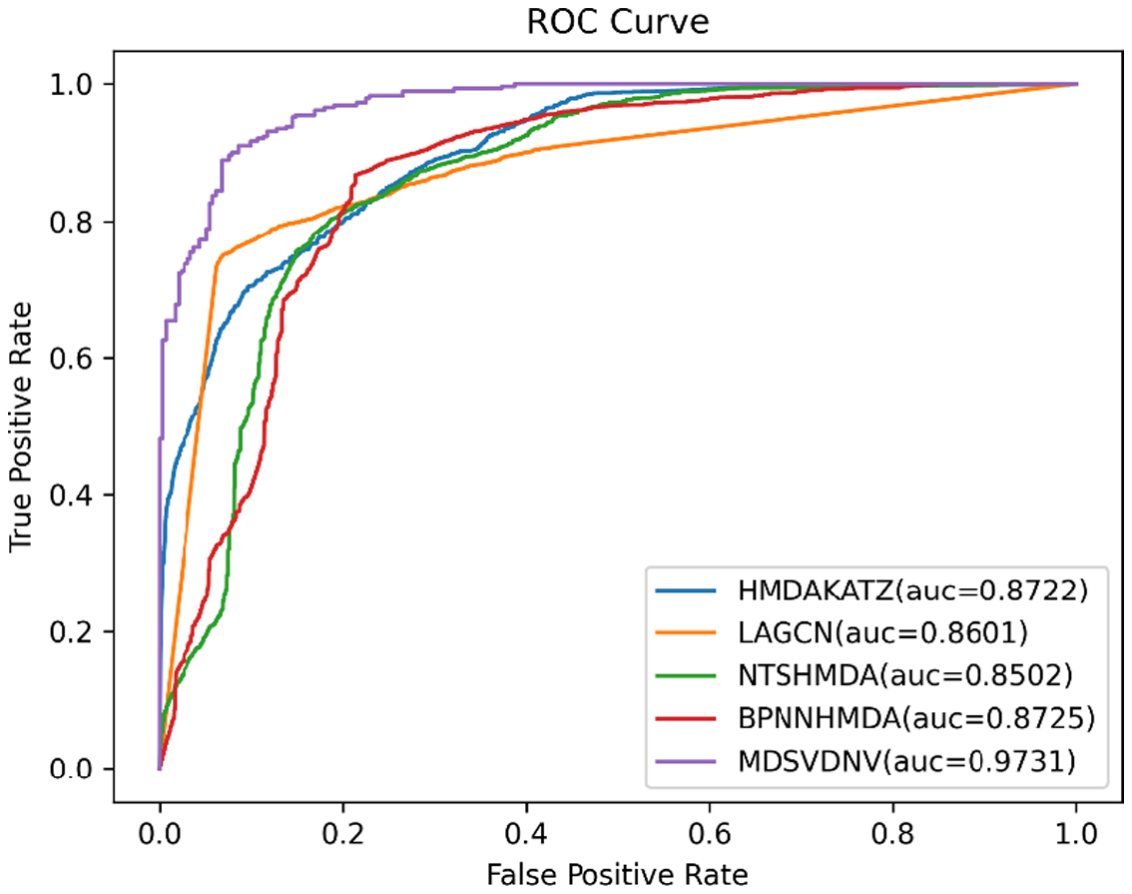
ROC curves of five competitive methods on aBiofilm.

**Figure 6:**
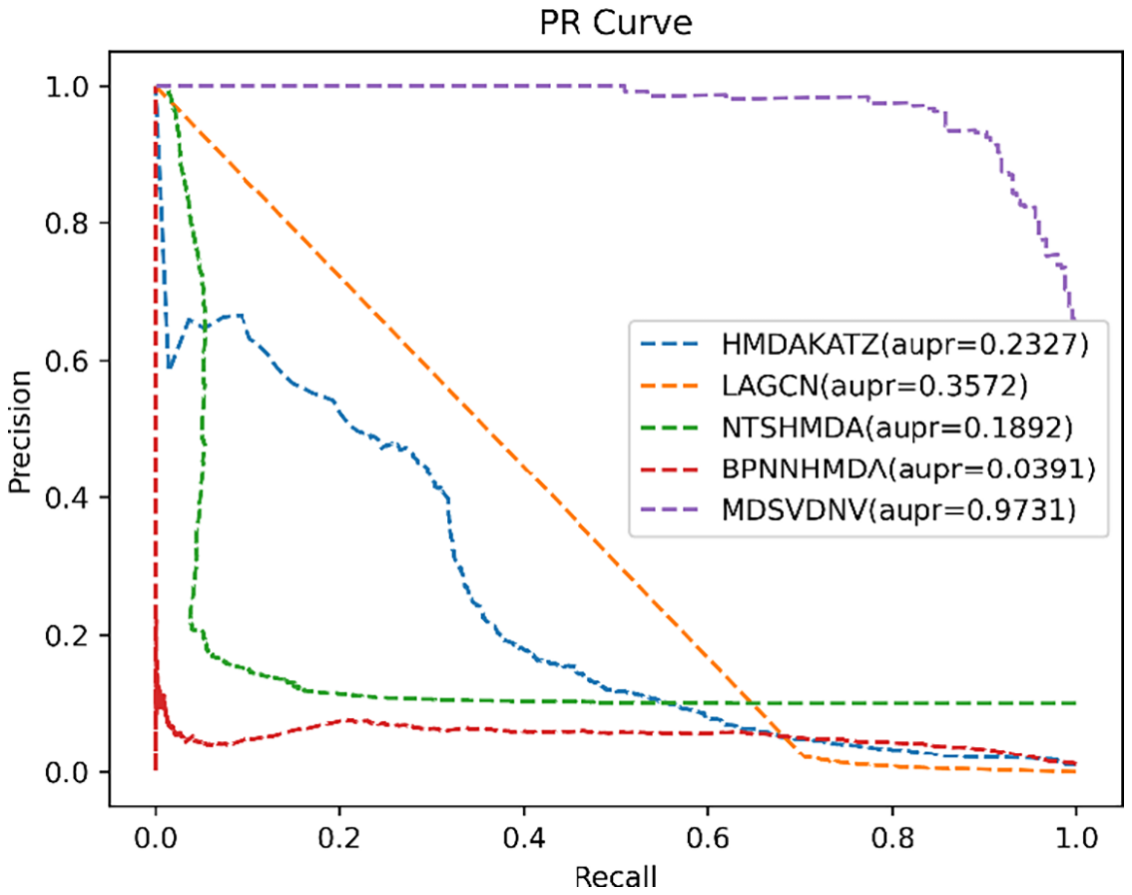
PR curves of five competitive methods on MDAD.

**Figure 7:**
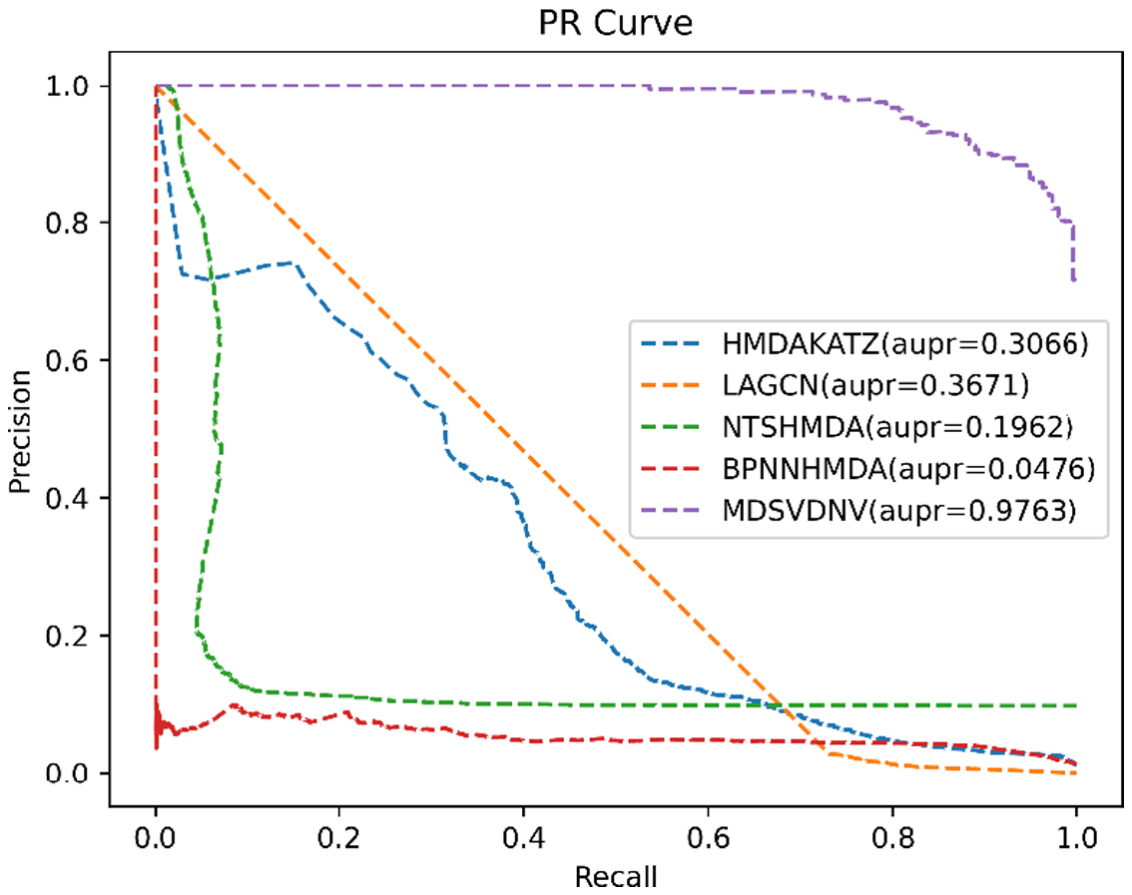
PR curves of five competitive methods on aBiofilm.

In order to visualize the advantages of the method, we drew ROC curves based on the two datasets as shown in Figures 4, 5 and PR curves based on the two datasets as shown in Figures 6, 7. It is obvious that MDSVDNV can achieve the highest AUC value of 0.9851 and the highest AUPRC value of 0.9893 under the 5-fold CV and MDAD/aBiofilm. Moreover, by combining linear and non-linear features in the training process, MDSVDNV obtained the highest average AUC value of 0.9703 as well. The results show that MDSVDNV is overall superior to all these competing methods.

### Ablation experiment

**Figure 8:**
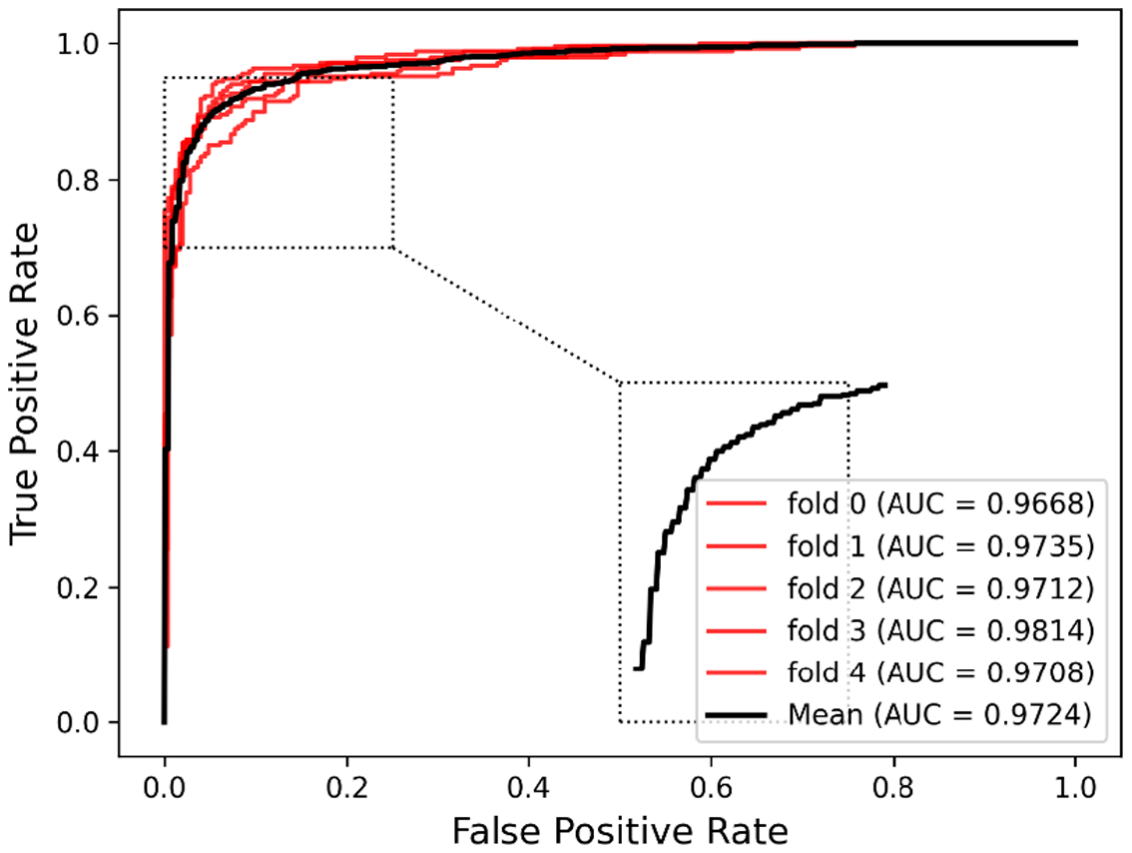
Prediction performance achieved by MDSVDNV under a 5-fold CV.

**Figure 9:**
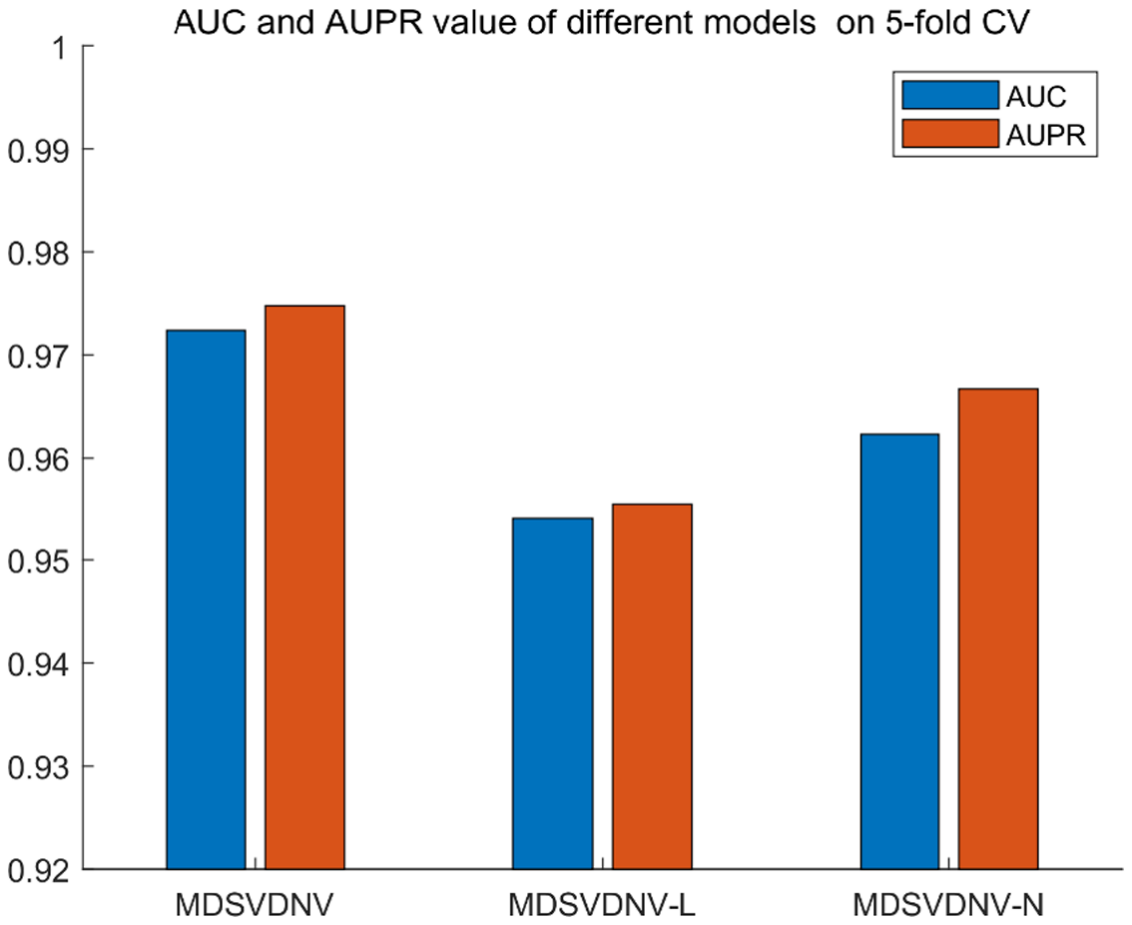
Performance comparison between MDSVDNV-L, MDSVDNV-N, and MDSVDNV.

Ablation experiments are conducted by systematically removing or modifying a component (a module) of an algorithm, model, or system to evaluate the effect of that component on overall performance. The purpose of ablation experiments is to study the contribution and role of a component of an algorithm, model, or system on performance in order to better understand how the algorithm, model, or system works. In order to anticipate the microbe–drug connection, we incorporate information from two different perspectives in this study. We conducted ablation experiments under a 5-fold CV to further confirm whether the combination of two linear and non-linear features favors the prediction power of the MDSVDNV model. The microbe–drug association prediction model using linear features is MDSVDNV-L, and the microbe–drug association prediction model using only non-linear features is MDSVDNV-N. The ROC curves of the prediction performance achieved by MDSVDNV at five times CV are plotted in Figure 8, and the comparison of the AUC and AUPRC performance of MDSVDNV-L, MDSVDNV-N, and MDSVDNV is shown in Figure 9. As can be seen in Table 2 and Figure 8, the highest AUC and AUPRC values of MDSVDNV reached 0.98, which proved that MDSVDNV has good overall performance. Table 3 and Figure 8 summarize the comparison of MDSVDNV and MDSVDNV- L with MDSVDNV-N. The AUC values of MDSVDNV- L, MDSVDNV - N, and MDSVDNV are 0.9724, 0.9540, and 0.8804, respectively. The AUPRC values of MDSVDNV- L, MDSVDNV- N, and MDSVDNV are 0.9748, 0.9555, and 0.8629, respectively. In different performance comparisons, MDSVDNV can achieve better results than MDSVDNV- L and MDSVDNV- N. The AUPRC values of MDSVDNV- L, MDSVDNV- N, and MDSVDNV are 0.9748, 0.9555, and 0.8629, respectively. In short, it is obvious that combining these two kinds of features can lead to better performance of MDSVDNV than models using only linear or non-linear features.

**Table 2:** Five-fold cross-validation results achieved by MDSVDNV.

	1	2	3	4	5	Average
AUC	0.9668	0.9735	0.9712	0.9814	0.9708	0.9724
AUPRC	0.9694	0.9765	0.9734	0.9840	0.9707	0.9748

**Table 3:** Comparison between MDSVDNV and its variants.

	MDSVDNV	MDSVDNV-L	MDSVDNV-N
AUC	0.9724	0.9540	0.9623
AUPRC	0.9748	0.9555	0.9667

### Case studies

We applied the MDSVDNV model to two widely used antimicrobial medications, *Ciprofloxacin* and *moxifloxacin*, as well as the microbe *Mycobacterium tuberculosis*, as our case studies in order to further assess the predictive efficacy of the method. The general procedure for each case study was as follows: First, the same number of negative samples and all microbe–drug association data were utilized to train the XGBoost classifier. After that, every microbe in the trial data that is not linked to the chosen drug is screened, and its feature vector is joined with the one for the present drug. All of these microbe–drug feature pairings are then sent through the trained classifier, and the output scores are used to calculate the likelihood that the microbe and drug will coexist. After ranking these scores in descending order, the top 20 microbe–drug associations were selected, and for the top 20 predicted microbes or drugs, we verified that the predicted microbe or drug associations were reported by searching the PubMed literature.

In terms of drugs, *Ciprofloxacin* is a fluoroquinolone antimicrobial agent used primarily for the treatment of most infectious diseases of tissues and body fluids caused by Gram-negative pathogens. More and more reports show that it has intimate relationships with a variety of human bacteria (Davis et al., 1996). *Ciprofloxacin*, for instance, has been shown by Gollapudi et al. to inhibit human immunodeficiency virus type 1 (HIV-1) (Gollapudi et al., 1998). Additionally, *Ciprofloxacin* has been shown by Hacioglu et al. to be effective against *Candida albicans (*Hacioglu et al., 2019*)*. *Enterococcus faecalis* was demonstrated by Kim and Woo to be a highly *Ciprofloxacin*-resistant bacterium (Kim and Woo, 2017). After everything was said and done, the findings revealed that 18 of the top 20 anticipated *Ciprofloxacin*-associated microbes could be supported by previously written research. A total of 90% of the time, MDSVDNV’s predictions were correct, suggesting that it may be somewhat useful for screening potential drug candidates in practical settings. The top 20 projected potential bacteria linked to *Ciprofloxacin* are listed in Table 4. Meanwhile, *moxifloxacin* is a fluoro antibacterial drug with notable effectiveness in the treatment of inflammatory disorders of the pelvis and the lungs (Balfour and Wiseman, 1999). *Moxifloxacin* is closely related to a variety of human bacteria, according to numerous studies. For instance, Villain and Dubois (2019) demonstrated the bactericidal efficacy of *moxifloxacin* against *Staphylococcus aureus*. It was discovered that moxifloxacin has anti-Candida abilities. *Moxifloxacin* has been shown to be a potent therapeutic option for *S. aureus* infections (Greimel et al., 2017). As shown in Tables 5, 18 of the top 20 candidate *moxifloxacin*-associated microorganisms were verified in previous literature.

**Table 4:** Top 20 predicted *Ciprofloxacin*-associated microbes.

Top	Drug	Microbe	Evidence
1	*Ciprofloxacin*	*Staphylococcus aureus*	PMID: 32488138
2	*Ciprofloxacin*	*Candida albicans*	PMID: 31471074
3	*Ciprofloxacin*	*Escherichia coli*	PMID: 33106267
4	*Ciprofloxacin*	*Clostridium perfringens*	PMID: 24944124
5	*Ciprofloxacin*	*Serratia marcescens*	PMID: 27052490
6	*Ciprofloxacin*	*Staphylococcus epidermis*	PMID: 10632381
7	*Ciprofloxacin*	*Streptococcus sanguis*	PMID: 11347679
8	*Ciprofloxacin*	*Streptococcus epidermidis*	PMID: 10632381
9	*Ciprofloxacin*	*Enterococcus faecalis*	PMID: 27790716
10	*Ciprofloxacin*	*Streptococcus*	PMID: 30502964
11	*Ciprofloxacin*	*Stenotrophomonas maltophilia*	PMID: 14982788
12	*Ciprofloxacin*	*Burkholderia cenocepacia*	PMID: 27799222
13	*Ciprofloxacin*	*Actinomyces oris*	Unconfirmed
14	*Ciprofloxacin*	*Morganella morganii*	PMID: 29942700
15	*Ciprofloxacin*	*Vibrio harveyi*	PMID: 27247095
16	*Ciprofloxacin*	*Plasmodium falciparum*	PMID: 31451506
17	*Ciprofloxacin*	*Candida* spp.	PMID: 30781782
18	*Ciprofloxacin*	*Klebsiella planticola*	PMID: 25465871
19	*Ciprofloxacin*	*Pichia anomala*	Unconfirmed
20	*Ciprofloxacin*	*Proteus vulgaris*	PMID: 34638966

**Table 5:** The top 20 predicted *Moxifloxacin*-associated microbes.

Top	Drug	Microbe	Evidence
1	*Moxifloxacin*	*Candida albicans*	PMID: 28409362
2	*Moxifloxacin*	*Pseudomonas aeruginosa*	PMID: 31691651
3	*Moxifloxacin*	*Staphylococcus aureus*	PMID: 31689174
4	*Moxifloxacin*	*Escherichia coli*	PMID: 31542319
5	*Moxifloxacin*	*Bacillus subtilis*	PMID: 30036828
6	*Moxifloxacin*	*Candida tropicalis*	PMID: 20455400
7	*Moxifloxacin*	*Haemophilus influenzae*	PMID: 11856249
8	*Moxifloxacin*	*Bacillus cereus*	PMID: 21834669
9	*Moxifloxacin*	*Human immunodeficiency virus 1*	Unconfirmed
10	*Moxifloxacin*	*Staphylococcus epidermis*	PMID: 11249827
11	*Moxifloxacin*	*Staphylococcus epidermidis*	PMID: 31516359
12	*Moxifloxacin*	*Mycobacterium avium*	PMID: 21353489
13	*Moxifloxacin*	*Citrobacter freundii*	PMID: 15992072
14	*Moxifloxacin*	*Eikenella corrodens*	PMID: 11897609
15	*Moxifloxacin*	*Neisseria gonorrhoeae*	PMID: 26603424
16	*Moxifloxacin*	*Listeria monocytogenes*	PMID: 28739228
17	*Moxifloxacin*	*Human herpesvirus 5*	Unconfirmed
18	*Moxifloxacin*	*Clostridium perfringens*	PMID: 29486533
19	*Moxifloxacin*	*Burkholderia pseudomallei*	PMID: 15731198
20	*Moxifloxacin*	*Actinomyces oris*	PMID: 26538502

Additionally, *Mycobacterium tuberculosis* is one of the microorganisms that we selected to employ for our case study. This *Gram-positive aerobic bacterium* may infect all bodily organs and is the source of tuberculosis, one of the deadliest diseases in the world. According to the 2019 Global Tuberculosis Report (WHO Global Tuberculosis Report, 2019), due to tuberculosis, 1.5 million people perished in 2018. Table 6 shows that 17 of the top 20 potential medicines for *Mycobacterium tuberculosis* are supported by previous research. In light of this, we can say that MDSVDNV exhibits satisfactory prediction ability in case studies involving both drugs and microbes.

**Table 6:** Top 20 predicted *Mycobacterium tuberculosis*-associated drugs.

Top	Microbe	Drug	Evidence
1	*Mycobacterium tuberculosis*	*Calanolide A*	PMID: 14980631
2	*Mycobacterium tuberculosis*	*Ceforanide*	PMID: 7624446
3	*Mycobacterium tuberculosis*	*Ciprofloxacin*	PMID: 16154314
4	*Mycobacterium tuberculosis*	*Rilpivirine*	Unconfirmed
5	*Mycobacterium tuberculosis*	*Pyrazinamide*	PMID: 26521205
6	*Mycobacterium tuberculosis*	*Vanillylacetone*	Unconfirmed
7	*Mycobacterium tuberculosis*	*Hydrogen peroxide*	PMID: 30551469
8	*Mycobacterium tuberculosis*	*Vitamin C*	PMID: 23695675
9	*Mycobacterium tuberculosis*	*Lopinavir*	PMID: 21442799
10	*Mycobacterium tuberculosis*	*Gentamicin*	PMID: 22143521
11	*Mycobacterium tuberculosis*	*Darunavir*	PMID: 28193650
12	*Mycobacterium tuberculosis*	*Minocycline*	PMID: 30597040
13	*Mycobacterium tuberculosis*	*Amikacin*	PMID: 29311078
14	*Mycobacterium tuberculosis*	*Tobramycin*	PMID: 19723387
15	*Mycobacterium tuberculosis*	*Zinc oxide*	PMID: 33845951
16	*Mycobacterium tuberculosis*	*Saquinavir*	PMID: 33841429
17	*Mycobacterium tuberculosis*	*Polysorbate 80*	Unconfirmed
18	*Mycobacterium tuberculosis*	*Vitamin E*	PMID: 26491981
19	*Mycobacterium tuberculosis*	*beta-Pinene*	PMID: 19753839
20	*Mycobacterium tuberculosis*	*Zidovudine*	PMID: 16154314

## Discussion and conclusion

Humans and microorganisms are interconnected and dependent on one another, according to clinical research. Predicting microbe–drug interactions can help with the development of microbe-derived treatments and drugs, which is crucial for the early detection, diagnosis, and treatment of disease. In this article, by combining the linear and non-linear features of drugs and microbes, we suggest a unique computational model, MDSVDNV, for predicting probable connections between microbes and drugs. The AUC and AUPRC values of MDSVDNV were higher than those of the five competitive prediction methods, which means that MDSVDNV may be a useful tool for the identification of potential microbial–drug associations and has the potential for pharmacological clinical treatments in the future. Moreover, MDSVDNV can be seen as an open framework in which more feature extraction methods can be applied flexibly. However, MDSVDNV also has certain limitations, which are mainly caused by the limitations of the datasets (e.g., heterogeneous network MDN) used in this study, and it is almost impossible to fully reflect the complex interactions between microbes and drugs by relying only on the relevant data. Meanwhile, Node2vec is unable to retain the rich and valuable information of different node types in the heterogeneous network, which will be improved by the expansion of the experimental data and the introduction of more advanced representation learning methods in future research.

## Data availability statement

The original contributions presented in the study are included in the article/supplementary material, further inquiries can be directed to the corresponding author.

## Author contributions

HT: Conceptualization, Methodology, Writing – original draft. ZZ: Resources, Supervision, Validation, Writing – review & editing. XL: Conceptualization, Software, Supervision, Writing – review & editing. YC: Data curation, Software, Visualization, Writing – original draft. ZY: Formal analysis, Software, Validation, Writing – original draft. LW: Funding acquisition, Investigation, Project administration, Supervision, Writing – review & editing, Writing – original draft.
